# Bridging the Gap: How to Get Osteopathic Residents Into Fellowships

**DOI:** 10.7759/cureus.27980

**Published:** 2022-08-13

**Authors:** Christina Colosimo, David R Mann, Sidra Bhuller, Douglas Opie, Zachary Beam, James Yon, J. Bracken Burns, Kristen Conrad-Schnetz

**Affiliations:** 1 Trauma Surgery, Cooper Medical School of Rowan University, Camden, USA; 2 General Surgery, Medical University of South Carolina, Charleston, USA; 3 General Surgery, Hospital Corporation of America (HCA) HealthONE Sky Ridge Medical Center, Lone Tree, USA; 4 Surgery, Mountain Vista Medical Center, Mesa, USA; 5 General and Trauma Surgery, Mercy Clinic, Springfield, USA; 6 General Surgery, Trauma Critical Care, New Hanover Regional Medical Center, Wilmington, USA; 7 Department of Surgery, East Tennessee State University, Johnson City, USA; 8 Department of Surgery, South Pointe Hospital Cleveland Clinic, Cleveland, USA

**Keywords:** fellowship match, american college of osteopathic surgeons, surgical subspecialty, fellowship, osteopathic fellow

## Abstract

Introduction

The fellowship match process is convoluted, with each specialty having its match on its timeline- with some programs having a Post Graduate Year (PGY) 4^th^-year or 5^th^-year match.

This study aims to identify tangible recommendations for osteopathic surgery residents to use to improve their applications and, ultimately, the success rate for matching into post-graduate fellowship training.

Methods

In October 2021, as a part of the American College of Osteopathic Surgeons (ACOS) Strategic Planning efforts, the ACOS Resident Student Section sent a questionnaire to the listed email contact for each surgical fellowship program. Fellowship coordinators and program directors were included in the survey. The programs that were included in the study were vascular, thoracic (which included cardiothoracic), surgical critical care, endocrine, hepatobiliary, transplant, pediatric, surgical oncology, breast, minimally invasive, and colorectal surgery.

Results

Of the 108 programs that answered the survey, 36% of them reported they currently had an osteopathic fellow, and another 29% said they had an osteopathic fellow in the past. 35% of the programs listed that they had never had an osteopathic fellow in their program.

In regards to how residents can improve their application for fellowship matches the most common answer was research in the field, they were trying to match into. They wanted to see high scores on the United States Medical Licensing Examination (USMLE) and American Board of Surgery In-Training Examination (ABSITE) exams. They also noted that they wanted candidates from more well know residency programs, where they knew the residents would have gotten good training.

Conclusion

We recommend that any potential fellowship applicant focus on the following three areas increase competitiveness for matching into fellowship training: publication in the desired field, increased overall scholarly activity, and increased ABSITE scores.

## Introduction

The Single Accreditation System (SAS) for graduate medical education (GME) was created in August 2014 when the American Osteopathic Association (AOA) and the American Association of Colleges of Osteopathic Medicine (AACOM) signed the memorandum of understanding with the Accreditation Council for Graduate Medical Education (ACGME) [1]. Before SAS, general surgery residents training in AOA-approved programs were not able to match into ACGME-approved Fellowship programs. 

ACGME is a single match, whereas the fellowship match process is more convoluted, with each specialty having its match on its timeline- with some programs having a Post Graduate Year (PGY) 4^th^-year or 5^th^-year match.

Previous studies have examined the effects of SAS on the match success of osteopathic candidates in surgical specialties. One study showed two characteristics were predominant in programs that accepted more osteopathic candidates: interviewing more candidates for first-year positions and reporting a higher percentage of female residents [2]. Another study identified three major recommendations for osteopathic students to become more competitive for successfully matching into surgical specialty residencies: increasing research, performing well on the United States Medical Licensing Exam (USMLE), and completing sub-internships in that field [3]. 

Currently, there is a paucity of data examining the key factors for improving the competitiveness of osteopathic surgery residents for matching into surgery specialty in the area of SAS. This study aims to identify tangible recommendations for osteopathic surgery residents to use to improve their applications and, ultimately, the success rate for matching into post-graduate fellowship training.

## Materials and methods

In October 2021, as a part of the American College of Osteopathic Surgeons (ACOS) Strategic Planning efforts, the ACOS Resident Student Section sent a questionnaire to the listed email contact for each surgical fellowship program. If a program coordinator and program director were listed, both were emailed. The programs that were included in the study were vascular, thoracic (which included cardiothoracic), surgical critical care, endocrine, hepatobiliary, transplant, pediatric, surgical oncology, breast, minimally invasive, and colorectal surgery. Eleven surgical specialty fellowships were included in the study. Of the 834 programs, 824 of them had listed contacts online. Emails were sent to the listed contacts separated into respective specialty groups. Three separate attempts were made by email from October 2021 to January 2022 to obtain the data. Responses were stored, and analysis was performed with Microsoft Excel (Microsoft® Corp., Redmond, WA).

The questionnaire requested answers to the following items: respondent name, specialty, program, and email. 

Participants were asked for responses to the following: (1) Do you have any osteopathic fellows in your program? If yes, how many? (2) Do you find osteopathic residents are equal to allopathic residents? (3) If the answers to either of the above questions are “no”, what can osteopathic residents do to be more competitive for your program? (This was an open-ended question.)

## Results

Of the 824 programs contacted, we had a response rate of approximately 13%, with 108 responses (Table 1). When looking at each survey, the highest reply rate was 27% for thoracic and hepatobiliary, and the lowest was 2% for minimally invasive surgery (Table 1).

**Table 1:** Response to the survey by specialty. SCC: Squamous cell carcinoma, HPB: Hepato-pancreatico-biliary, MIS: Multisystem inflammatory syndrome

Specialty	Programs	Missing contacts	Replied to survey	Percentage that replied to survey
Vascular	186	2	18	10%
Thoracic	56		15	27%
SCC	126	5	20	17%
Endocrine	25		6	24%
HPB	15		4	27%
Transplant	64	3	9	15%
Pediatric	56		6	11%
Surgical Oncology	36		6	17%
Breast	60		8	13%
MIS	154		3	2%
Colorectal	56		13	23%
Total	834	10	108	13%

Table 2 shows the number of osteopathic fellows in each specialty for all respondents. Each specialty has at least one osteopathic fellow currently in it, with surgical critical care reporting the most osteopathic fellows (11). Of the 108 programs that answered the survey, 36% of them reported they currently had an osteopathic fellow, and another 29% said they had an osteopathic fellow in the past. Thirty-five percent of the programs listed that they had never had an osteopathic fellow in their program. 

**Table 2:** Osteopathic fellows in fellowships as reported by programs by region. SCC: Squamous cell carcinoma, HPB: Hepato-pancreatico-biliary, MIS: Multisystem inflammatory syndrome

Specialty	Yes	No	Past	Total responses
Vascular	8	6	4	18
Thoracic	5	8	2	15
SCC	11	3	6	20
Endocrine	1	2	3	6
HPB	1	1	2	4
Transplant	3	6	0	9
Pediatric	2	3	1	6
Surgical Oncology	2	3	1	6
Breast	2	1	5	8
MIS	2	0	1	3
Colorectal	2	5	6	13
	39	38	31	108

The majority of programs (82%) wrote that osteopathic candidates were equivalent to allopathic candidates. Only one program wrote that osteopathic candidates were not equal to allopathic candidates. Ten programs stated they never had an osteopathic fellow and were unable to assess if they were equivalent. Six programs commented that there was variability in osteopathic training and one commented that there was a difference in their depth of knowledge and performance on exams. 

**Figure 1:**
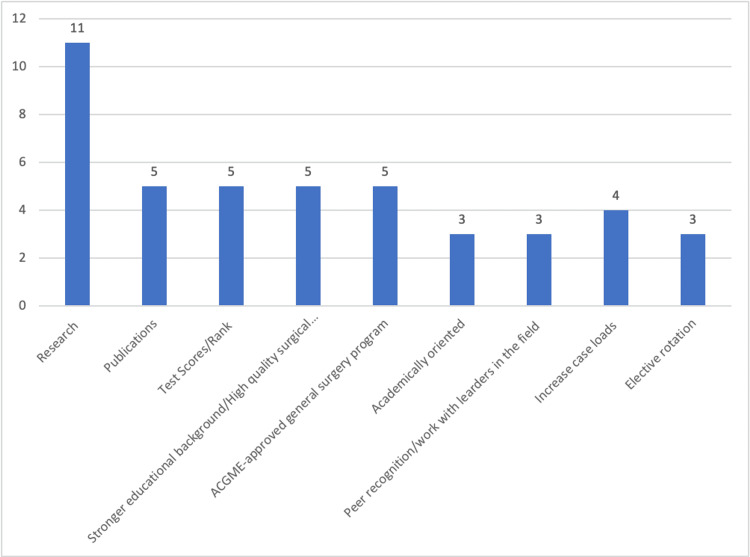
How can osteopathic residents become better candidates?

In regards to how residents can improve their application for fellowship matches the most common answer was research in the field, they were trying to match into (Figure 1 ). Another five programs explicitly stated they wanted to see publications from the candidates, not just research. They wanted to see high scores on USMLE and ABSITE exams. They also noted that they wanted candidates from more well know residency programs, where they knew the residents would have gotten good training. Five programs were said to be ACGME accredited programs even though we are now in the SAS. A few programs wrote they were recruiting academically oriented fellows and wanted to read that in their statement. The rest of the suggestions, which were self-explanatory, are to increase residency caseload, work with leaders in the field and have them write letters of recommendation, as well as do elective rotations at programs residents want to match into. 

## Discussion

Osteopathic fellowships were originally established as a result of the limited acceptance of osteopathic residents into ACGME accredited fellowships [4]. Now with the SAS, there still exists some continued gap in knowledge about osteopathic residents. This may attribute to the decreased acceptance of osteopathic residents into fellowship. As we saw in our survey, despite all programs now falling under ACGME, there were still several fellowships asking for candidates from accredited programs. Osteopathic residents that graduated during the SAS still have the choice to be accredited by the American Board of Surgery (ABS) or the American Osteopathic Board of Surgery (AOBS), but they all are ABS board eligible [5]. There were also comments coming from a more well-known program that osteopathic training was variable. Despite this response, a six-year review of allopathic and osteopathic general surgery residency applications at one training program found that the groups were not different in the number of letters of recommendation, volunteer activities, scholarly works, and advanced degrees [6]. However, they did observe that osteopathic USMLE step one scores were higher in comparison. 

Osteopathic general surgery residents still represent the minority [7], and this is one explanation for the inconsistencies seen in the responses of the program directors. There was a recent study that compared university-based osteopathic vs. allopathic surgeons, which found that osteopathic university attendings represented the minority [8]. They saw across ranks and specialties; osteopathic surgeons had fewer publications and citations. Their recommendation was to foster mentors and research development in medical school and residency to alleviate these discrepancies. One identified explanation for this was that the majority of allopathic attendings came from university residencies, while the majority of osteopathic attendings came from community-based residences. 

Currently, 80% of general surgery residents apply for fellowship [9]. This has been increasing from previous decades. One study found the reason for the push toward fellowship is for a better lifestyle, market share differentiation, prestige, and not wanting to pursue general surgery as a career [10]. While some fellowships are required for credentialing to perform specialized surgery (cardiothoracic, transplant, pediatric, etc.), some fellowships are more for comfort in the specialized area of colorectal, hepatobiliary, minimally invasive surgery, etc. 

There have been several studies that look at what is required to match into different fellowships. A study that looked into successful matriculation into colorectal fellowship found that U.S. citizens, allopathic residents, and the number of programs an applicant applied to were more associated with a successful match [11]. Another study that looked at matching into pediatric surgery fellowship showed higher chances of matching for those who attended a residency program with a pediatric surgery fellowship, were in a program with dedicated research years, came from a university program, had higher ABSITE scores, came from an allopathic medical school and had more publications (median 12) [12,13]. For surgical oncology, one survey study of program directors identified benchmarks to receive an interview. These included ABSITE scores of 50% or greater and first-author publications with a mean of two [14]. However, the biggest determinant for those who matched was the quality of the interview. 

Physician survey response rates are low [15], as we saw in our study. One explanation given in the study is higher response rates for topics of high interest. Some specialties were probably less interested in this topic. A study was able to change their response rate from 19.6% to 64.8% with $25 incentives [16]; however, this was not feasible for our study. Response rates across specialties were not uniform, and this could lead to selection bias. This would affect the number of programs reporting DOs previously or currently training in their program. There also could have been a response bias that encouraged the participants to answer osteopathic residents were equal to allopathic residents. 

## Conclusions

We recommend that any potential fellowship applicant focus on these three areas increase competitiveness for matching into fellowship training: publication in the desired field, increased overall scholarly activity, and increased ABSITE scores. Other areas recommended were to increase residency case volume when able to, work with leaders in the field, have them write letters of recommendation, and do elective rotations at programs where they want to match at. 
